# Endothelial dysfunction, carotid artery plaque burden, and conventional exercise-induced myocardial ischemia as predictors of coronary artery disease prognosis

**DOI:** 10.1186/1476-7120-6-61

**Published:** 2008-12-16

**Authors:** Bonpei Takase, Yoshihiro Matsushima, Akimi Uehata, Masayuki Ishihara, Akira Kurita

**Affiliations:** 1 National Defense Medical College Research Institute, Division of Biomedical Engineering, Saitama, Japan; 2 Department of Cardiovascular Medicine, Course of Medical and Dental Sciences, Graduate School of Biomedical Sciences, Nagasaki University, Nagasaki, Japan; 3 Self Defense Forces Central Hospital, Tokyo, Japan

## Abstract

**Background:**

While both flow-mediated vasodilation (FMD) in the brachial artery (BA), which measures endothelium-dependent vasodilatation, and intima-media thickness (IMT) in the carotid artery are correlated with the prognosis of coronary artery disease (CAD), it is not clear which modality is a better predictor of CAD. Furthermore, it has not been fully determined whether either of these modalities is superior to conventional ST-segment depression on exercise stress electrocardiogram (ECG) as a predictor. Thus, the goal of the present study was to compare the predictive value of FMD, IMT, and stress ECG for CAD prognosis.

**Methods and Results:**

A total of 103 consecutive patients (62 ± 9 years old, 79 men) with clinically suspected CAD had FMD and nitroglycerin-induced dilation (NTG-D) in the BA, carotid artery IMT measurement using high-resolution ultrasound, and exercise treadmill testing. The 73 CAD patients and 30 normal coronary patients were followed for 50 ± 15 months. Fifteen patients had coronary events during this period (1 cardiac death, 2 non-fatal myocardial infarctions, 3 acute heart failures, and 9 unstable anginas). On Kaplan-Meier analysis, only FMD and stress ECG were significant predictors for cardiac events.

**Conclusion:**

Brachial endothelial function as reflected by FMD and conventional exercise stress testing has comparable prognostic value, whereas carotid artery plaque burden appears to be less powerful for predicting future cardiac events.

## Background

Endothelial function is a sensitive barometer of arterial health and can reflect the status of atheroma such as the presence of vulnerable and/or stable plaque. Several studies have suggested that endothelial vasomotor function testing, such as brachial artery flow-mediated vasodilatation (FMD), has prognostic value [1-6].

Furthermore, the carotid atheroma burden has also been reported to be well correlated with coronary artery disease severity [7-9]. Some studies show that carotid artery intima-media thickness (IMT) predicts a future major coronary event, including acute coronary syndrome[10]. In contrast, there are other reports showing that the carotid plaque burden is not correlated with adverse coronary artery disease (CAD) outcomes and instead predicts cerebrovascular events[11]. Exercise stress testing is not only used as a conventional diagnostic modality but is also used as a prognostic tool to predict adverse outcomes in patients with known CAD[12]. A prognostic study comparing endothelial dysfunction and carotid artery plaque burden has been done. However, there are few studies that have directly compared the relatively new diagnostic modalities (FMD and/or IMT) with conventional exercise stress testing.

Thus, the purpose of this study was to determine the relative prognostic importance of brachial artery endothelial function as determined by FMD, the carotid atheroma burden as determined by IMT, and exercise-induced myocardial ischemia in patients with suspected CAD.

## Methods

### Study populations

A total of 103 consecutive patients who were referred to the Division of Cardiology at the National Defense Medical College due to clinically suspected coronary artery disease and who were subsequently admitted for cardiac catheterization and agreed with study protocol were included in this study. Suspected coronary artery disease was defined as having either typical angina symptoms or atypical chest pain with exercise-induced myocardial ischemia by exercise testing. The patients were enrolled in this study from August 1999 to February 2001. The study population consisted of 79 men and 24 women, whose ages ranged from 35 to 80 years (mean age, 62 ± 9 years). Exclusion criteria were as follows; the patients present acute illness including acute coronary syndrome, uncompensated phase of chronic illness such as chronic renal failure, chronic heart failure and chronic lung disorders. The patients in whom percutaneous coronary intervention was performed at the time of coronary angiography was included and this percutaneous coronary intervention was not considered as cardiac events. None of the patients had electrocardiogram (ECG) abnormalities (such as bundle-branch block) on resting ECG that would have precluded measurement of ischemic ST or T-wave changes, nor did any patient have ECG evidence of Wolff-Parkinson-White syndrome or left ventricular hypertrophy. The presence of a Q wave > 0.04 sec duration in a lead, and the presence of baseline ST-segment morphology, defined as a depression or an elevation of 1 mm or more as compared to the isoelectric line, were considered for ST-segment depression measurements. Oral medications, including antianginal agents, were discontinued for at least five half-lives prior to FMD, IMT, and exercise testing, but sublingual nitroglycerin was administered during acute anginal episodes. All subjects were aware of the investigative nature of the study and gave their informed consent. The research protocol was approved by the local institutional ethics committee.

### Ultrasound measurements of brachial artery FMD and carotid artery IMT

All ultrasound studies were done at approximately 08:00 in the morning in a temperature-controlled room (25°C) with the fasting subject resting in the supine position. Heavy meals, including a high-fat meal and caffeine-containing beverages, were prohibited from the night before the study. During the ultrasound procedures, blood pressure and heart rate were recorded from the left arm every 3 min using an automatic sphygmomanometer (Nihon Korin, BP-203, Tokyo, Japan). A 7.5 MHz high-resolution ultrasound (Hewlett-Packard, SONOS 2000, Andover, MA, USA) was used to measure carotid artery IMT, using essentially the same method as previously reported[13,14]. IMT measurements were obtained using longitudinal projections approximately 2 cm below the bifurcation at the starting point of the bulbus. Despite recent guideline[15], IMT was defined as maximum thickness of IMT at the region of interest detected in both left and right carotid artery including common carotid artery.

FMD was measured using a previously validated technique[16,17]. Brachial artery diameter and flow velocity were imaged using a 7.5 MHz high-resolution ultrasound (Hewlett-Packard, SONOS 2000). After baseline measurements of brachial artery diameter and flow velocity, a small-width blood pressure cuff (Hokanson SC-10, Seattle, WA, USA) was inflated on the proximal portion of the forearm to occlusive pressure (200 mmHg) for 5 min in order to induce reactive hyperemia. The FMD was expressed as the percent change in the diameter relative to the baseline diameter at rest. The average peak velocity was obtained from the pulsed Doppler signals recorded at rest and immediately after cuff deflation. The flow volume in the brachial artery was calculated by multiplying the average peak velocity and the vessel cross-sectional area. Reactive hyperemia was defined as the relative increase in the brachial blood flow calculated as the maximal flow measured immediately after cuff deflation divided by the flow obtained at rest (baseline).

Finally, after a 15 min interval, nitroglycerin-induced vasodilation (NTG-D) was obtained because we put a 15 min interval in our institute to make sure the canceling the effect of reactive hyperemia despite guideline[15,15]. Baseline measurements of brachial artery diameter and flow velocity were again obtained, and then 0.3 mg of sublingual nitroglycerin was administered. Three minutes later, the brachial artery diameter was recorded. The NTG-D was defined as the percent change of the brachial artery diameter relative to the baseline diameter. All images were recorded on super VHS videotape for later analysis. All ultrasound studies and parameter measurements were done by one of the investigators who was blinded to the patient's clinical information. A previous study in our laboratory showed that the intra- and inter-observer variabilities (coefficient of variation) for repeated measures of diameter before and after reactive hyperemia in the brachial artery were < 3%[17] As previously reported[17], all brachial diameter images are videotaped and off line review were performed. After cuff deflation, brachial artery images were reviewed for at least 3 min, maximum dilation that occurred were approximately 78 sec (averaged time) after cuff deflection and the brachial artery diameter for FMD was obtained at these points (maximum dilation in each patient).

### Exercise testing

Exercise treadmill testing was performed to assess exercise-induced myocardial ischemia using the Bruce or modified Bruce protocol. Exercise was continued until the heart rate reached 85% of the estimated maximum age-predicted target heart rate for each patient. If a patient could not exercise to the target heart rate, the patient continued to exercise until exhaustion or until cardiac symptoms, such as precordial discomfort, dyspnea, or palpitations, occurred. Throughout the study, 12-lead ECGs were monitored continuously and recorded at one min intervals using a Marquette CASE 12 (Marquette Electronics, Inc., Milwaukee, WI, USA) and blood pressure was measured by Korotkoff's method at 1-minute interval. ST-segment depression was measured 80 ms after the J point. A horizontal or downsloping ST-segment depression of at least 1.0 mm was considered significant for exercise-induced ischemia. Stress ECGs were interpreted visually by expert cardiologists blinded to the clinical information and any clinical parameters, including the FMD and IMT measurements. Exercise treadmill testing was performed within two days of the FMD and IMT studies.

### Coronary angiographic evaluation

Coronary angiography was conducted using the standard Judkins femoral approach method. Coronary angiographic findings were interpreted visually by at least three expert cardiologists blinded to the results of the FMD and IMT measurements. Significant coronary artery disease was defined as the presence of vessel luminal diameter stenosis > 70% in a major branch of the coronary arteries.

### End point and follow-up

Clinical long-term follow-up was done and confirmed by reviewing death certificates, hospital records, telephone conversations with the local treating physicians, the patients, or the patients' relatives. During follow-up, the time to the occurrence of cardiac events was assessed. Cardiac events were defined as cardiac death, myocardial infarction, and hospital readmission for unstable angina and acute heart failure due to documented ischemia such as the presence of significant ischemic ECG changes. Myocardial infarction was defined as new ST elevations (> 0.1 mV) in 2 contiguous leads or an elevation of creatine kinase levels > 2 times the upper limit of normal. Since aspirin, β-blocking agents, angiotensin-converting enzyme (ACE) inhibitors, and statins might affect either endothelial vasodilator function or disease progression, the use of these medical therapies was documented during follow-up. The clinical long-term follow up analyses were performed in the entire population as well as CAD group only.

### Statistics

All data are presented as the means ± SD and a 95% confidence interval (CI) or frequency (%), unless otherwise stated. The baseline clinical characteristics of the groups were compared using the two-tailed Student's *t-*test for continuous variables and the chi-square or Fisher's exact test for non-continuous variables, as appropriate. Survival was determined using Kaplan-Meier curves, and outcomes were compared using the log-rank test. A receiver operating characteristic (ROC) curve was used to acquire appropriate sensitivity and specificity of FMD, IMT and stress ECGs for the diagnosis of adverse outcome during follow up period. Multiple logistic regression analysis was performed to identify the independent variables for predicting cardiac events. In order to determine the predictive value of FMD, IMT, and stress ECG, multiple logistic regression analysis for all the cardiac events was applied to these variables and they were compared with the other factors including conventional clinical variables. Baseline clinical characteristics, such as coronary risk factors, the information on follow-up medication, the number of coronary artery diseased vessels, the presence of the baseline revascularization procedures, and left ventricular ejection fractions, were included as independent variables. Univariate predictors which had P < 0.1 were included into multiple logistic regression analysis. All of these statistical analyses were performed using SPSS version 11.0 (SPSS Japan Inc., Tokyo, Japan). Statistical significance was set at the P < 0.05 level.

## Results

The clinical characteristics of the study population are summarized in Table 1. There were 73 patients with significant coronary artery disease (CAD group) and 30 patients without significant coronary artery stenosis (NCAD group). In NCAD group, 20 out 30 patients had none significant coronary stenosis in the major branch of coronary arteries. Patients in the CAD group were significantly older, were more frequently male, and had a higher prevalence of active smoking, hypertension, diabetes mellitus, hyperlipidemia, and lower HDL levels than in the NCAD group. Approximately half of the patients in the CAD group had multi-vessel disease. Among CAD group, 26 patients were performed elective percutaneous coronary intervention and these percutaneous coronary interventions were not included as cardiac events. In addition, this study population revealed that there were no patients with markedly impaired left ventricular function such as left ventricular ejection fraction < 40%. The use of medical therapies described in the method section are also shown in Table 1.

**Table 1:** Clinical characteristics of study populations

	CAD group (n = 73)	NCAD group(n = 30)
Age, years	64 ± 10*	58 ± 11
Men	60 (82)*	11 (34)
Body-mass index, kg/m^2^	24.0 ± 4.2	21.9 ± 3.9
Current smoker	49 (67) *	13 (43)
Hypertension	50 (68) *	14 (47)
Diabetes mellitus	32 (44) *	4 (13)
Hyperlipidemia	55 (75) *	16 (53)
Family history of coronary artery disease	17 (19)	5 (17)
Total cholesterol (mg/dl)	202 ± 33	209 ± 109
Triglyceride (mg/dl)	162 ± 70	170 ± 109
HDL cholesterol (mg/dl)	48 ± 13*	57 ± 13
Fasting blood sugar (mg/dl)	114 ± 37	101 ± 21
Uric acid (mg/dl)	6.0 ± 1.4	6.2 ± 1.5
No. coronary diseased vessels		
1	38(52)	0
2	25(34)	0
3	10(14)	0
Follow up medical therapies		
ACE-I or ARB	24(33)*	4(13)
Beta-blockers	47(64)*	7(23)
Calcium channel blockers	38(52)	11(37)
Statins	39(53)*	8(27)
Nitrates	44(60)*	7(23)

Data are expressed as the mean ± SD and numbers in parenthesis expresses percentage of prevalence; CAD group, patients with significant coronary artery disease; NCAD group, patients with non-significant coronary artery stenosis; HDL, high density lipoprotein; Hypertension, blood pressure ≥ 160/95 mmHg; Diabetes mellitus, fasting blood sugar ≥ 126 mg/dl; Hyperlipidemia, total cholesterol ≥ 230 mg/dl; ACE-I or ARB, angiotensin-converting enzyme inhibitors or angiotensin II receptor blockers; No., numbers; * P < 0.05 vs. NCAD group.

### Brachial artery FMD, carotid artery IMT, and stress ECG

The heart rate, blood pressure, baseline brachial artery diameter, blood flow in the brachial artery, and reactive hyperemia were not significantly different between the CAD and NCAD groups (Table 2). FMD was significantly lower in the CAD group than in the NCAD group, while there was no difference in NTG-D between the two groups. The mean IMT values and exercise testing ST-segment depression were significantly higher in the CAD group than in the NCAD group. There was no abnormal blood pressure response to exercise in any patients (systolic blood pressure > 220 mmHg and/or diastolic blood pressure > 110 mmHg).

**Table 2:** The summary of ultrasound study measurements for flow-mediated vasodilation in the brachial artery and intimal media thickness in the carotid artery as well as exercise testing

	CAD group (n = 73)	NCAD group (n = 30)
Heart rate at baseline (beats/min)	66 ± 7	58 ± 11
Systolic blood pressure at baseline (mmHg)	125 ± 17	119 ± 13
Diastolic blood pressure at baseline (mmHg)	73 ± 10	70 ± 13
Brachial artery diameter at baseline (mm)	4.32 ± 0.64	4.21 ± 0.83
Brachial artery blood flow at baseline (ml/min)	64.5 ± 35.0	68.5 ± 37.3
Reactive hyperemia	4.4 ± 2.7	4.8 ± 3.1
Flow-mediated vasodilation (%)	3.7 ± 3.0*	7.5 ± 2.9
Nitroglycerin-induced vasodilation (%)	13.5 ± 4.3	15.7 ± 4.9
Intimal media thickness in carotid artery (mm)	1.1 ± 0.2*	0.7 ± 0.1
Exercise testing ST segment depression (mm)	1.84 ± 0.99*	0.77± 0.8

Data are expressed as the mean ± SD; CAD group, patients with significant coronary artery disease; NCAD group, patients with non-significant coronary artery stenosis; * P < 0.05 vs. NCAD group.

### Long-term follow-up and brachial artery FMD, carotid artery IMT, and stress ECG

The mean follow-up period was 50 ± 15 months. During this period, 15 patients suffered from cardiac events: 1 cardiac death, 2 non-fatal myocardial infarctions, 3 acute heart failures, and 9 unstable anginas (Table 3). Among these 15 patients, one NCAD group patient had frequent angina attacks at rest that required readmission. This patient was initially labeled as having unstable angina. However, on coronary angiography, no significant stenosis was seen; since acetylcholine-provoked vasospasm was documented, the patient was diagnosed as having vasospastic angina. The remaining 14 patients had CAD on baseline coronary angiography. Of these 14 patients, 6 developed new coronary lesions, and 9 received revascularization procedures (1 coronary bypass surgery and 8 percutaneous coronary interventions).

**Table 3:** List of patients with cardiac events during follow-up period

	Age (y/o)	Sex	CAD (+/-)	Type of event	Revascularization procedures	Time to event (months)
	53	M	+	Unstable angina	PCI	3
	60	M	+	Myocardial infarction	Medical treatment	48
	77	F	+	Acute heart failure	Medical treatment	6
	76	M	+	Unstable angina	PCI	43
	46	M	+	Acute heart failure	Medical treatment	14
	69	M	+	Unstable angina	CABG	2
	84	F	+	Unstable angina	PCI	43
	45	M	+	Myocardial infarction	PCI	12
	69	M	+	Acute heart failure	Medical treatment	2
	59	F	-	Unstable angina*	Medical treatment	34
	63	M	+	Unstable angina	PCI	58
	71	M	+	Unstable angina	PCI	10
	74	F	+	Unstable angina	PCI	11
	54	M	+	Unstable angina	PCI	51
	76	M	+	Fetal Myocardial infarction		11

No. number; y/o, years old; M, male: F, female; CAD (+/-), presence or absence of coronary artery disease; NL, no significant coronary artery disease; *, subsequently and newly diagnosed as vasospastic angina pectoris; PCI, percutaneous coronary intervention; CABG, coronary bypass surgery.

**Figure 1:**
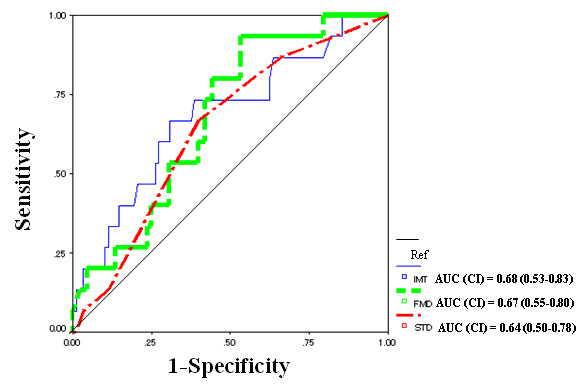
A receiver operating characteristic (ROC) curve of intima media thickness (IMT) in the carotid artery, flow-mediated vasodilation (FMD) in the brachial artery and ST-segment changes during exercise treadmill testing (STD) for the diagnosing significant coronary artery disease. ROC curve revealed that areas under curve (AUC; CI, confidence intervals) in IMT, FMD and STD were not significantly different. Ref., reference line.

Compared to patients without cardiac events, patients with cardiac events had significantly impaired FMD and more severe exercise-induced myocardial ischemia (Table 4). In addition, patients with cardiac events tended to have higher IMT, though this difference was not statistically significant. However, when ROC curves for detecting adverse outcome of cardiac events were compared, areas under the curve (AUC) of IMT, FMD and exercise-induced myocardial ischemia (expressed as STD in Figure 1) were not significantly different as shown in Figure 1.

**Table 4:** The summary of ultrasound study measurements for flow-mediated vasodilation in the brachial artery and intimal media thickness

	With cardiac events (n = 15)	Without cardiac events (n = 88)
Heart rate at baseline (beats/min)	66 ± 6	67 ± 5
Systolic blood pressure at baseline (mmHg)	125 ± 16	124 ± 17
Diastolic blood pressure at baseline (mmHg)	74 ± 14	72 ± 11
Brachial artery diameter at baseline (mm)	4.57 ± 0.90	4.21 ± 0.62
Brachial artery blood flow at baseline (ml/min)	69.4 ± 36.6	64.9 ± 35.3
Reactive hyperemia	4.3 ± 2.1	4.5 ± 2.9
Flow-mediated vasodilation (%)	3.0 ± 2.1*	5.2 ± 3.7
Nitroglycerin-induced vasodilation (%)	14.1 ± 4.4	14.1 ± 4.6
Intimal media thickness in carotid artery (mm)	1.1 ± 0.3	0.9 ± 0.2
Exercise testing ST segment depression (mm)	1.60 ± 1.18*	0.93 ± 0.86

In the carotid artery as well as exercise testing; Comparison between patients with and without cardiac events.

Data are expressed as the mean ± SD; * P < 0.05 vs. Without cardiac events.

**Figure 2:**
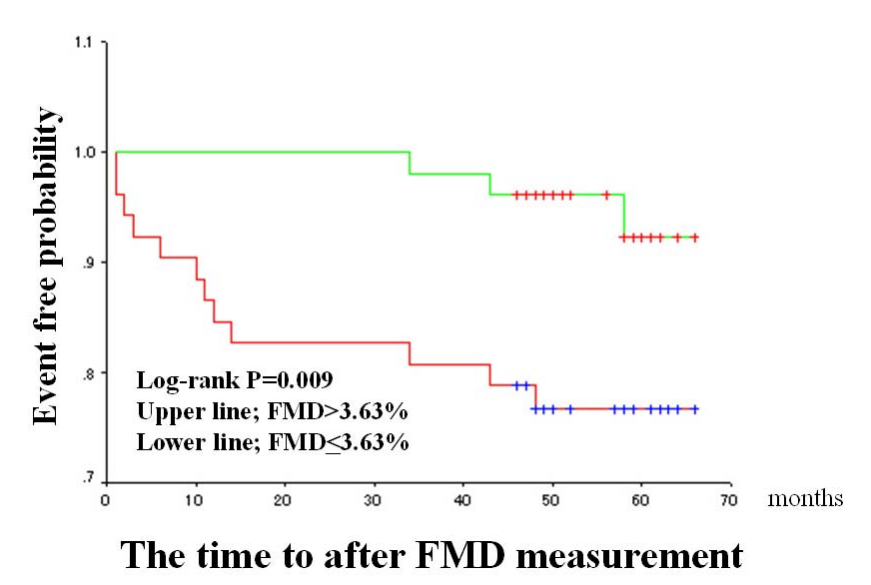
Flow-mediated vasodilatation (FMD) in the brachial artery and cardiac events in the entire population. Kaplan-Meier curve shows the cumulative proportion of patients with cardiac events during follow-up. Patients were divided into two groups based on a median FMD value of 3.63%.

**Figure 3:**
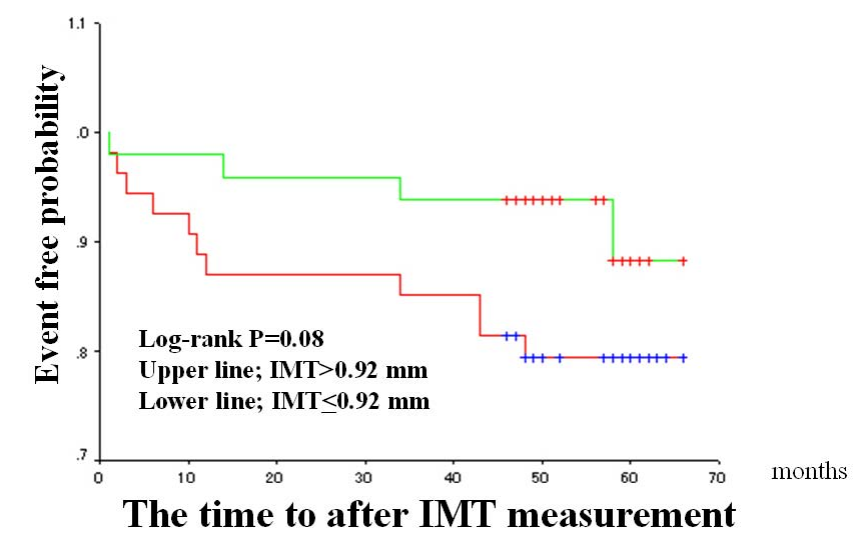
Intima-media thickness (IMT) in the carotid artery and cardiac events in the entire population. The format is the same as for Figure 2. Patients were divided into two groups based on a median IMT value of 0.92 mm.

**Figure 4:**
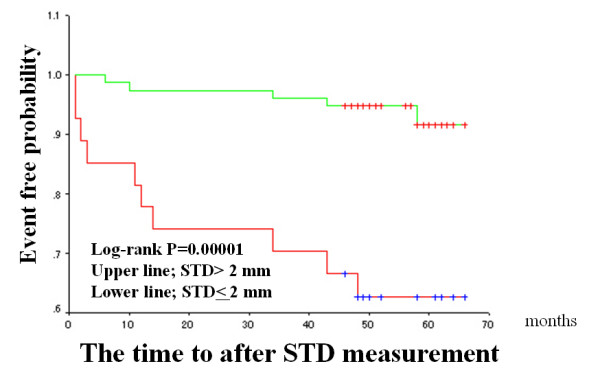
Exercise-induced ischemia (ST-segment depression > 2 mm) and cardiac events in the entire population. The format is the same as for Figure 2. Patients were divided into two groups based on an STD value of > 2 mm. STD, ST-segment depression 80 ms after the J point.

The median FMD and IMT values were used as arbitrary the cut-off values (median value of the patients studied), and Kaplan-Meier cardiac event-free survival curves were compared (Figures 2 to 4). Patients with impaired FMD had a significantly higher cardiac event rate. However, patients with higher IMT values tended to have a poor prognosis, though this trend did not reach statistical significance. When a cut-off value of > 2.0 mm was used on stress ECG and the Kaplan-Meier curve was analyzed, patients with severe exercise-induced myocardial ischemia had a significantly higher cardiac event rate than those without exercise-induced myocardial ischemia (Figure 4).

**Figure 5:**
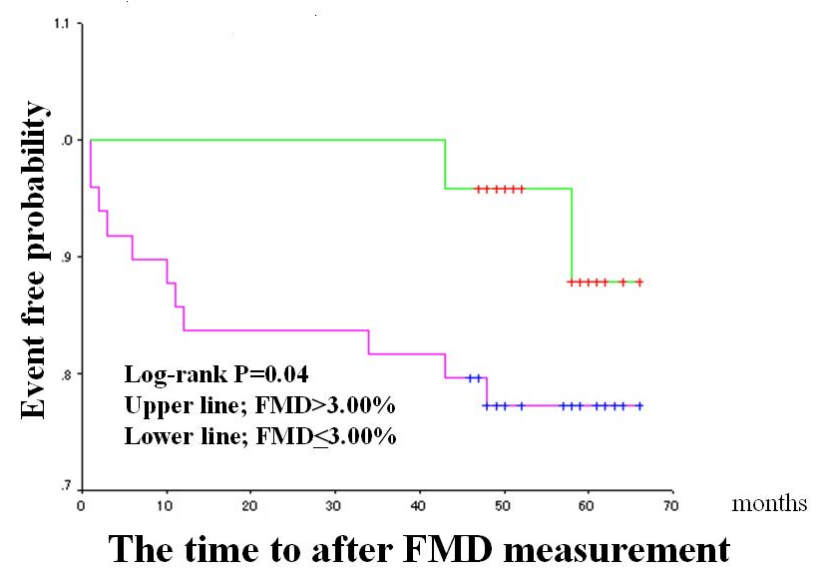
Flow-mediated vasodilatation (FMD) in the brachial artery and cardiac events in CAD group. Kaplan-Meier curve shows the cumulative proportion of patients with cardiac events during follow-up. Patients were divided into two groups based on a median FMD value of 3.00%.

**Figure 6:**
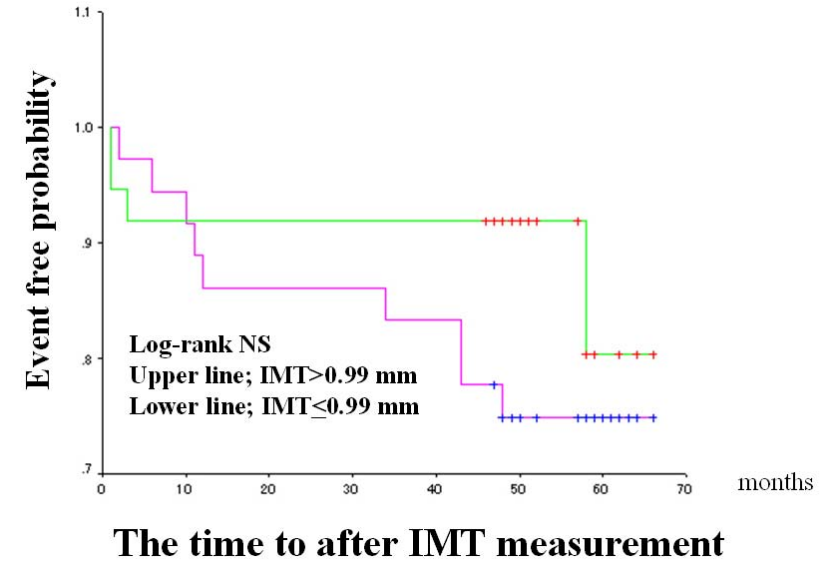
Intima-media thickness (IMT) in the carotid artery and cardiac events in CAD group. The format is the same as for Figure 2. Patients were divided into two groups based on a median IMT value of 0.99 mm.

**Figure 7:**
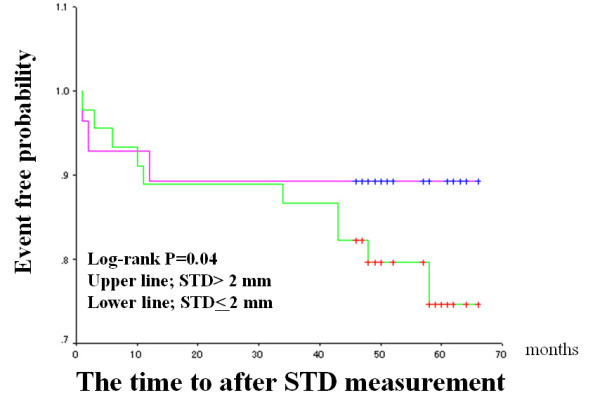
Exercise-induced ischemia (ST-segment depression > 2 mm) and cardiac events in CAD group. The format is the same as for Figure 2. Patients were divided into two groups based on an STD value of > 2 mm. STD, ST-segment depression 80 ms after the J point.

The same Kaplan-Meier cardiac event-free survival curves were compared in CAD group only (Figures 5 to 7). As result, the similar findings to the entire population were observed. Patients with impaired FMD had a significantly higher cardiac event rate. But, patients with higher IMT values have no significant adverse outcome. Again, the patients with severe exercise-induced myocardial ischemia had a significantly higher cardiac event rate.

In order to compare the predictive value of FMD, IMT, and stress ECG with conventional clinical variables, we did a multiple logistic regression analysis for all the cardiac events. Baseline clinical characteristics, such as coronary risk factors, the number of coronary artery diseased vessels, the presence of the baseline revascularization procedures, and left ventricular ejection fractions, were included as independent variables. The information on follow-up medication was also included. We found that the univariate predictors for cardiac events included: the number of diseased coronary arteries, diabetes mellitus, HDL-cholesterol level, FMD, IMT, and stress ECG. However, FMD, IMT, and stress ECG were the only multivariate predictors for cardiac events (Table 5).

**Table 5:** Independent predictors for all cardiac events in multiple logistic regression analysis

Independent variables	Odds ratio	Confidence intervals	P values
Flow-mediated vasodilation of brachial artery	0.74	0.53–0.99	P < 0.05
Intimal media thickness in carotid artery	1.20	1.01–2.30	P < 0.05
Exercise testing ST segment depression	1.85	1.04–3.30	P < 0.05

## Discussion

The main findings in our results demonstrate that brachial artery endothelial function (FMD) and conventional exercise stress testing have comparable prognostic power for predicting adverse outcome in patients with suspected CAD. However, the carotid atheroma burden as reflected by IMT does not strongly predict cardiac events. Event-free survival analyses showed that impaired FMD and marked exercise-induced myocardial ischemia were significantly correlated with increased cardiac events, whereas increased IMT tended to be associated with increased cardiac events, but this trend was not statistically significant.

Since the endothelium is the major regulator of vascular homeostasis, endothelial function could be a very sensitive barometer of the functional state of atheroma. It is known that endothelial function rapidly responds to pro-atherogenic or anti-atherogenic stimuli [18-20]. The endothelium is involved in vasodilation, suppression of smooth muscle cell growth, and inhibition of the inflammatory response. Endothelial dysfunction plays an integral role in almost all stages of atherosclerosis. Thus, FMD is associated with rapid progression of atherosclerosis and poor cardiovascular prognosis, which explains its predictive power for cardiac events. Therefore, endothelial vasomotor function testing, such as FMD, may have significant value in clinical practice.

Exercise-induced myocardial ischemia is related to both functional and morphological atherosclerotic changes. The pathophysiology of exercise-induced myocardial ischemia has been extensively investigated[21,22]. Fixed coronary atherosclerosis, as well as vasomotion during exercise, is associated with exercise-induced myocardial ischemia[23]. Endothelial function is one of the factors that contributes to this vasomotion[24]. Profound ischemic ECG change (ST segment depression > 2 mm) is a well-known prognostic indicator for cardiac events[12,23]. Our results agree with these reports.

However, we could not determine why the carotid atheroma burden, as reflected by IMT, did not predict cardiac events. Since IMT represents a morphological change, dynamic function testing, such as FMD or exercise testing, theoretically does not reflect the IMT. Some studies show that IMT is more predictive of stroke than of cardiac events[11], while IMT is reported to be correlated with the severity of coronary artery sclerosis[9]. Given our small sample size, it is possible that there might not have been enough power for the Kaplan-Meier analysis to predict cardiac events. However, our data show that increased IMT tended to reduce cardiac event-free survival, and multiple logistic regression analysis supported the possible prognostic role of IMT. As shown in Table 2, although the contribution of FMD and IMT towards CAD progression is not identical, both FMD and IMT were well correlated with the severity of coronary atherosclerosis. These findings are in agreement with previous studies[25,26]. The diagnostic power for CAD and the ability to predict prognosis is sometimes dissociated in the clinical setting. Thus, even though IMT may predict prognosis, IMT might not be as clinically useful for prognostication as it is for making the diagnosis of CAD.

Study limitations: This study had several limitations. First, medications were discontinued before the FMD and IMT studies, as well as before exercise treadmill testing. This is the definitive limitation when our study findings are applied to the clinical practice. Since FMD and exercise testing may be affected by various medications, the therapeutic impact on the relationship among FMD, IMT, and exercise testing with respect to CAD prognostication should be investigated in future studies. Second, we did not use an exercise diagnostic score, such as the Duke exercise scoring system[21,22] which includes not only ECG changes but also the exercise tolerance time and other exercise parameters. However, the simplest and standard prognostic ECG parameter (ST segment depression > 2 mm) was a predictor of cardiac events in this study. Thus, ECG analysis may be sufficient for comparing FMD, IMT, and exercise testing. Third, the number of patients studied was relatively small; this study should be repeated with a larger sample size to confirm these findings. Forth, we analyzed Kaplan-Meier cardiac event-free survival curves in both the entire population and the CAD group. The entire population seems to be heterogeneous in terms of the presence of significant coronary stenosis. However, most of the study patients, 93 out of 103, showed none significant but at least 25% stenosis on the major branch of coronary arteries and the remaining 10 patients did not received intravascular ultrasound (IVUS) study so that the presence of atheroma plaque could not be totally excluded in these patients. Since the acute coronary syndrome could possibly occur from none significant coronary artery stenosis, we examined Kaplan-Meier cardiac event-free survival curves in the entire population. In addition, Kaplan-Meier cardiac event-free survival curves in the CAD group showed similar findings in this study. Fifth, the FMD of manual ultrasonically assessment of brachial artery endothelial function is highly operator-dependent and time consuming procedure so that the improved equipment of FMD measure should be developed and our findings were confirmed in the near future. Recently, we developed semi-automatic FMD measurement system that is not so much operator dependent[27]. We could use this new equipment in next study. Sixth, even if IMT was significant predictor for cardiac events by multiple logistic regression analysis, Kaplan-Meier curve showed marginal statistical power (P = 0.08, Figure 3) for separating patients with and without cardiac events. According to previous report[7], IMT could reflect the severity of CAD. This lack of statistical power might be explained by the fact that the number of patients studied was not large enough to detect the role of IMT. Lastly, the numbers of hard cardiac events such as cardiac death was very small in this study (approximately 1%). This might contribute the conflicting results of prognostic power of FMD among the previous reports. Among them, if the hard cardiac events rate were high, FMD did not predict these events (around 10% events rate of these report)[28,29]. In contrast, Frick et al report (around 1% hard cardiac events) showed that FMD is still the predictor for cardiac events. In our study, rate of hard cardiac event was about 1% so that our study agrees with that of Frick et al[30]. In general, the prognosis of CAD is tended to be better in Asian country than in western country. We speculate that conflicting evidence on the prognostic power of FMD might depend on the different and/or heterogeneous population studied.

Notwithstanding all these limitations, the data suggest that endothelial dysfunction, carotid atheroma burden, and exercise stress testing appear to reflect prognosis in patients with suspected CAD. However, our results indicate that brachial endothelial function, as reflected by FMD, and conventional exercise stress testing are comparable with respect to prognostic value, whereas carotid artery plaque burden might be less powerful for predicting future cardiac events, even though it may still be a possible prognostic predictor.

## Competing interests

The authors declare that they have no competing interests.

## Authors' contributions

YM carried out the patient enrollment and each parameter's measurement. AU, MI and AK participated in the design of the study. BT conceived of the study, and participated in its design and coordination.
